# Multi-Trajectories of Intrinsic Capacity Decline and Their Impact on Age-Related Outcomes: A 20-Year National Longitudinal Cohort Study

**DOI:** 10.14336/AD.2023.1115-1

**Published:** 2023-11-21

**Authors:** Lin-Chieh Meng, Hui-Min Chuang, Wan-Hsuan Lu, Wei-Ju Lee, Chih-Kuang Liang, Ching-Hui Loh, Fei-Yuan Hsiao, Liang-Kung Chen

**Affiliations:** ^1^Graduate Institute of Clinical Pharmacy, College of Medicine, National Taiwan University, Taipei, Taiwan.; ^2^Gerontopole of Toulouse, Institute of Ageing, Toulouse University Hospital (CHU Toulouse), Toulouse, France.; ^3^Maintain Aging Research Team, CERPOP, Inserm, Université Paul Sabatier, Toulouse, France.; ^4^Center for Healthy Longevity and Aging Sciences, National Yang Ming Chiao Tung University, Taipei, Taiwan.; ^5^Center for Geriatrics and Gerontology, Taipei Veterans General Hospital, Taipei, Taiwan.; ^6^Department of Family Medicine, Taipei Veterans General Hospital Yuanshan Branch, Yi-Lan County, Taiwan.; ^7^Center for Geriatrics and Gerontology, Kaohsiung Veterans General Hospital, Kaohsiung City, Taiwan.; ^8^Center for Healthy Longevity, Hualien Tzu Chi Hospital Buddhist Tzu Chi Medical Foundation, Hualien County, Taiwan.; ^9^Department of Pharmacy, National Taiwan University Hospital, Taipei, Taiwan.; ^10^School of Pharmacy, College of Medicine, National Taiwan University, Taipei, Taiwan.; ^11^Taipei Municipal Gan-Dau Hospital (Managed by Taipei Veterans General Hospital), Taipei, Taiwan.

**Keywords:** healthy aging, intrinsic capacity, functional limitation, falls, quality of life, mortality

## Abstract

The existence of intrinsic capacity (IC) subtypes and their distinct impacts on age-related outcomes remain unexplored. This study sought to investigate IC impairment trajectories across domains and their associations with the risk of age-related outcomes, including falls, functional limitations, reduced quality of life (QoL) and mortality at 4- and 8-year follow-ups. The study sample comprised 1,782 older adults residing in the community from the Taiwan Longitudinal Study on Ageing (TLSA). Utilizing group-based multitrajectory modeling, distinct subtypes of IC decline trajectories across various domains were identified. Cox proportional hazard models and multivariable logistic regression analyses were employed to assess the associations between the identified subtypes and age-related outcomes. We identified four subtypes of IC decline: robust with mild decline (n=902), hearing loss with cognitive decline (n=197), physio-cognitive decline (PCD) with depression (n=373), and severe IC decline (n=310). Over the 4-year study period, compared to the robust with mild decline group, hearing loss with cognitive decline group exhibited a significantly higher risk of diminished QoL (OR=2.53 [1.66-3.86], p<0.01), whereas those in the PCD with depression group experienced an elevated risk of falls (OR=1.62 [1.18-2.23], p<0.01), as well as functional limitation (OR=2.61 [1.81-3.75], p<.01). Individuals in the severe IC decline group faced a substantially increased risk of all outcomes of interest. Distinct subtypes of IC decline across different domains have varying impacts on age-related outcomes, highlighting the need for a personalized approach to promote healthy ageing at the population level, while further investigation into specific pathophysiological mechanisms is warranted as well.

## INTRODUCTION

Population ageing is a global challenge, intensified by the increasing number of older individuals and extended periods of disability. The World Health Organization (WHO) has released the World Report on Ageing and Health, defining healthy ageing as the process of developing and maintaining functional ability to promote well-being in older age (apps.who.int/iris/handle/10665/355618). The paradigm of healthy ageing emphasises the significance of functional ability and intrinsic capacity (IC) as fundamental metrics in cultivating a life-course framework aimed at averting disability and dementia [1]. IC, which encompasses an individual's physical and mental capacities and can serve as an indicator of functional reserve during the ageing process, has prompted a shift in healthcare approach from the traditional disease-centric model to a function-centric paradigm within the framework of healthy ageing [2]. Normally, an individual's IC peaks in early adulthood and subsequently undergoes a gradual decline over time [1]. Given the significant impact that diminishing IC can have on functional capacity and overall well-being, it is crucial to continuously monitor and support IC throughout the ageing process.

In the WHO Integrated Care for Older People (ICOPE) framework, IC comprises five domains: locomotion, sensory (vision and hearing), vitality, psychological well-being, and cognition (apps.who.int/iris/handle/10665/325669). Previous studies have demonstrated the associations between impaired IC domains and various unfavorable outcomes, such as disability [3-5], falls [4, 5], hospitalizations [3], and mortality in older adults [4]. In addition to the examination of impairment within individual IC domains, several studies have delineated the associations between composite IC scores and age-related outcomes [3, 6]. Although these domains may signify distinct aetiologies either individually or compositely, their clustering also uncovers common pathophysiological pathways underlying the decline of IC in the ageing process. A recent cross-sectional study employing latent class analysis identified diverse patterns of IC impairments, characterized by distinct clinical features and negative outcomes within subtypes [7]. The study additionally revealed a strong correlation between impairments in locomotion and cognitive domains, supporting the existence of a physio-cognitive decline syndrome (PCDS) phenotype within the ageing process, wherein older adults without disability and dementia experience concurrent impairments in physical and cognitive function [8]. These findings highlight the heterogeneity of the ageing process and underscore the need for longitudinal investigations to elucidate the mechanisms driving IC decline. To promote healthier ageing, it is crucial to explore trajectories of IC decline while considering the clustering effects of different domains.

Within the purview of healthy ageing, healthcare practitioners and healthcare systems should consider various factors that contribute to declines in IC and functional capacity, such as multimorbidity, lifestyle, social determinants, and various other influences. Moreover, they should actively facilitate personalized health management strategies to foster the holistic well-being of older individuals [9, 10]. Consequently, ascertaining the prognostic significance of distinct subtypes of IC decline in relation to clinical outcomes assumes paramount precedence. In addition, examining the possible cluster effect of IC declines on an individual's quality of life (QoL), which encompasses social, mental, emotional and physical well-being, is also of great significance. Hence, this study aims to investigate the associations of IC declines subtypes with falls, functional limitation, reduced QoL and mortality through a 20-year longitudinal cohort survey and provide potential evidence on the pathophysiological mechanisms of IC decline to inform the design of personalized intervention programs for promoting healthy ageing.

## MATERIALS AND METHODS

### Data source and study subjects

**Figure 1.**
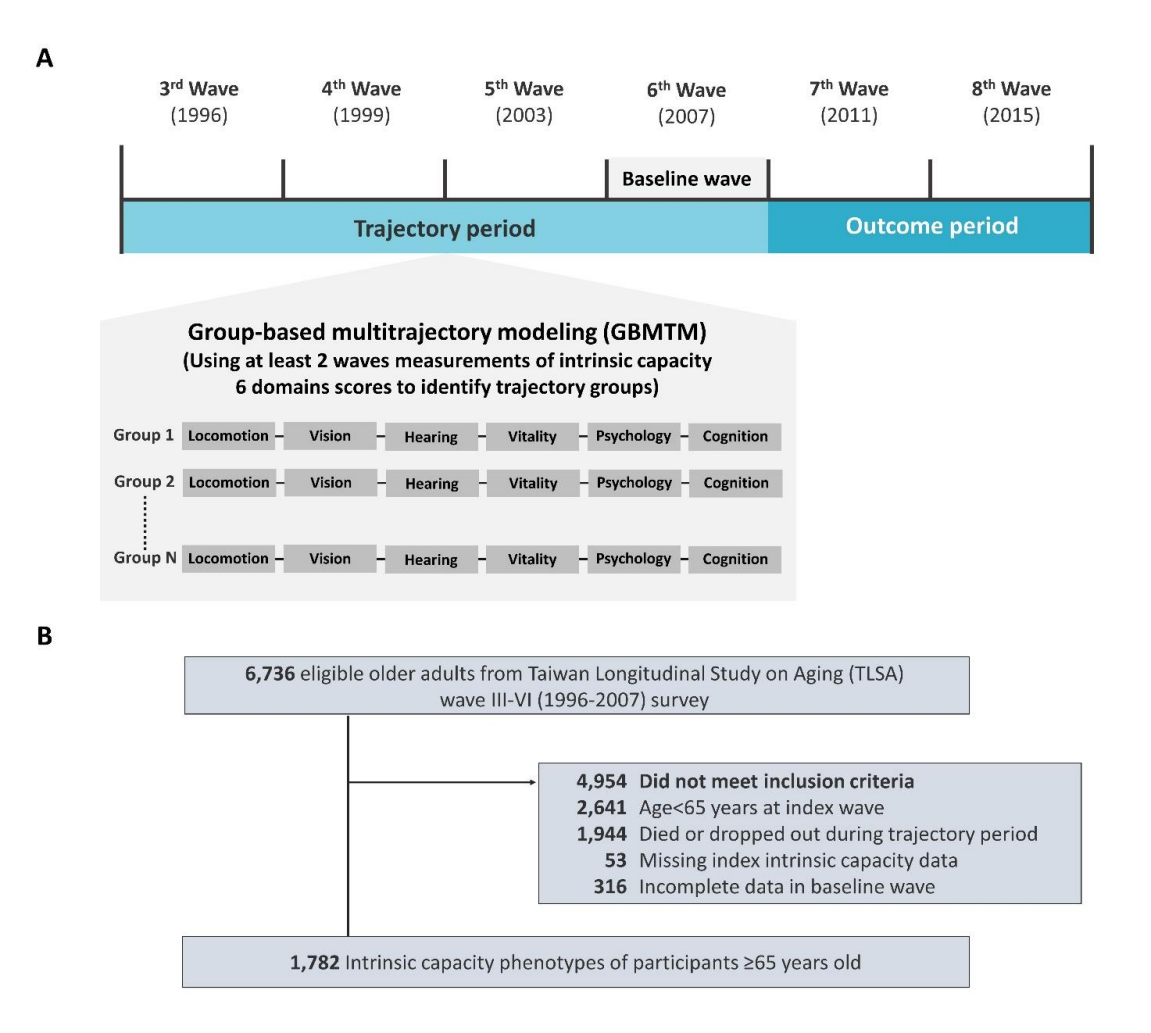
**Study design and study population selection. (A)** Scheme of study design **(B)** Inclusion and exclusion criteria for study participants.

The data employed in this investigation were derived from the Taiwan Longitudinal Study on Ageing (TLSA), a research project conducted by the Health and Welfare Data Science Center under the Ministry of Health and Welfare. The TLSA is a comprehensive nationwide survey that has been ongoing since 1989, with subsequent follow-ups conducted at intervals of 3-4 years. Notably, the survey has achieved an impressive response rate of up to 90%. The inception of the survey was initiated through collaboration between the Taiwan Health Promotion Administration (HPA) and the University of Michigan. Data collection was facilitated through in-person household interviews, encompassing assessments of demographic characteristics, household details, and evaluations of physical, functional, and mental health statuses of middle-aged and older adults. Further information regarding the TLSA can be accessed via the official website of the Taiwan Health Promotion Administration (www.hpa.gov.tw/1077/6197/e), as well as previous scholarly investigations [10, 11]. The study cohort consisted of individuals aged 65 years or older, drawn from the third (year 1996) to the sixth (year 2007, baseline wave of this study) waves of the TLSA survey. Participants who died or discontinued their participation during the trajectory period (n=1944), had incomplete data in any of the IC domains during the index wave (n=53), or exhibited incomplete data in the baseline wave (6^th^ wave, year 2007, n=316), were thus excluded from the analysis (Fig. 1).

### Measurement of IC

The five-domain IC was defined based on WHO ICOPE guidelines, comprising locomotion, sensory, vitality, psychological well-being, and cognition. The locomotion domain was assessed as in the Nagi questionnaire [12]. Participants who had difficulty or were unable to walk a distance of 200-300 m were considered to have limited mobility, i.e., impairment in the locomotion domain [11, 13]. The sensory domain was subdivided into vision and hearing. Visual acuity was assessed by asking participants, "How well can you see things (even with glasses or contact lenses)?" [14, 15], and those who answered "poor" or "very poor" were classified as having visual impairment. Hearing ability was evaluated by self-reported hearing impairment. Those who reported difficulties hearing in either ear (even with a hearing aid) would be classified as impaired hearing [15, 16]. In the vitality domain, appetite loss was used to represent nutritional status. Participants who had a poor appetite in the previous week were classified as malnutrition [7, 13]. Two questions from The Center for Epidemiologic Studies Depression (CES-D) questionnaire: “I felt that everything I did was an effort”, and “I could not get going” were used to measure the psychological domain [13, 17]. Participants answered “often” or “most of the time” to either question were considered to have an impairment in psychological domain. The cognition domain was evaluated by two questions from the Short Portable Mental Status Questionnaire (SPMSQ) regarding spatial and time orientation. Failing in either item was defined as an impairment in the cognition domain [6, 7].

### Outcomes of interest

This study examined several outcomes that serve as key indicators of healthy ageing. These outcomes included physical function limitation, impaired mobility performance, cognitive decline, falls, reduced QoL, and all-cause mortality. Physical function was assessed using the 6-item Activities of Daily Living (ADLs) and the 6-item Instrumental Activities of Daily Living (IADLs) scales [18, 19]. A decline in ADL or IADL scores of ≥1 point drop from baseline to follow-up survey was considered indicative of functional limitation. Mobility performance was evaluated using the 9 mobility activities, and a decline in scores of ≥1 point from baseline to follow-up survey was considered as a decline in mobility performance [10]. Cognitive function assessment included cognitive and memory tasks derived from the Short Portable Mental Status Questionnaire (SPMSQ). Higher scores indicated better cognitive function, and a decline of ≥ 1 point from baseline to follow-up survey was considered as cognitive decline [10]. Falls were assessed by self-reported fall events through interviews and questionnaires during the follow-up visits [20]. QoL was evaluated by the Life Satisfaction Index developed by Neugarten and colleagues [21]. The 10 questions were rated as yes (1) or no (0), with total scores ranging from 0 to 10. Reduced in QoL was defined as a decline of at least one point on the QoL at follow-up survey compared with baseline wave. All-cause mortality was assessed from the 2007 TLSA survey until Dec 2015. The date of death was identified from the National Death Registry held by the Ministry of Health and Welfare, Taiwan, which was linked with the study database [6]. Pre-planned analyses were conducted to assess the impact of trajectories in IC decline, by different domains, on 4- and 8-year decline in ADLs and IADLs, reduced mobility performance, cognitive decline, falls, reduced QoL, and mortality.

### Other variables

Demographic data included subjects’ age, sex, education level, smoking status, and alcohol use. Trained interviewers used a questionnaire to assess the QoL score, the SPMSQ score, and the CES-D score. Functional ability was also measured by ADL and IADL. Comorbidities were documented according to self-reported physician diagnosis, including hypertension, diabetes mellitus, hyperlipidaemia, heart disease, stroke, cancer, chronic obstructive pulmonary disease (COPD) or asthma, arthritis, gastric ulcer, hepatobiliary disease, cataract, chronic kidney disease, gout, and osteoporosis. Recognizing that multimorbidity is a well-established risk factor for disability and various age-related outcomes, we further computed the disease count to capture the potential impacts of multimorbidity.

### Statistical Analysis

Descriptive statistics were employed to analyze the demographic and clinical characteristics of all participants. Continuous variables in both the text and tables were presented as mean ± standard deviation, while categorical variables were expressed as percentages. The prevalence of impairments in IC domains during the trajectory period was assessed.

#### Phase 1: Group-based multi-trajectory modelling to distinguish IC decline subtypes

In the initial phase, we employed the group-based multi-trajectory model (GBMTM), a finite-mixture modelling approach, to identify distinct clusters of individuals exhibiting similar trajectories across multiple indicators over time. This facilitated the identification of groups of subjects displaying distinctive phenotypic presentations [22, 23]. Each of the six components representing impairments in IC were treated as time-varying binary variables in each wave throughout the trajectory period. To determine the most suitable model, we assessed the optimal number of trajectories and trajectory shapes following recommended procedures [17, 23, 24]. The selection of the most appropriate model was based on the Bayesian information criterion (BIC) value, which allowed for the comparison of models with different numbers of groups and trajectory shapes. For inclusion, each trajectory group had to consist of more than 5% of participants and have an average posterior probability (AvePP) value exceeding 0.7. The model with the highest BIC value was considered the optimal choice.

#### Phase 2: The association between IC decline subtypes and adverse outcomes

Baseline characteristics of different IC decline subtypes were compared using analysis of variance (ANOVA) for continuous variables, and chi-square tests or Fisher's exact test for categorical variables. Cox proportional hazard models and multivariate logistic regression models were utilized to assess the associations between IC decline subtypes and outcomes related to healthy ageing, as well as all-cause mortality with appropriate adjustments. The Kolmogorov-Type Supremum test was employed to verify the proportional hazards assumption for Cox proportional hazard models. Hazard ratios (HR) with 95% confidence intervals (CI) were reported for mortality, while adjusted odds ratios (aORs) with 95% CI were reported for falls, functional limitation, cognitive decline and reduced QoL. All statistical analyses were conducted using SAS, version 9.4 (SAS Institute Inc., Cary, NC) (26-28). A two-tailed p-value of ≤0.05 was considered statistically significant.

## RESULTS

**Figure 2.**
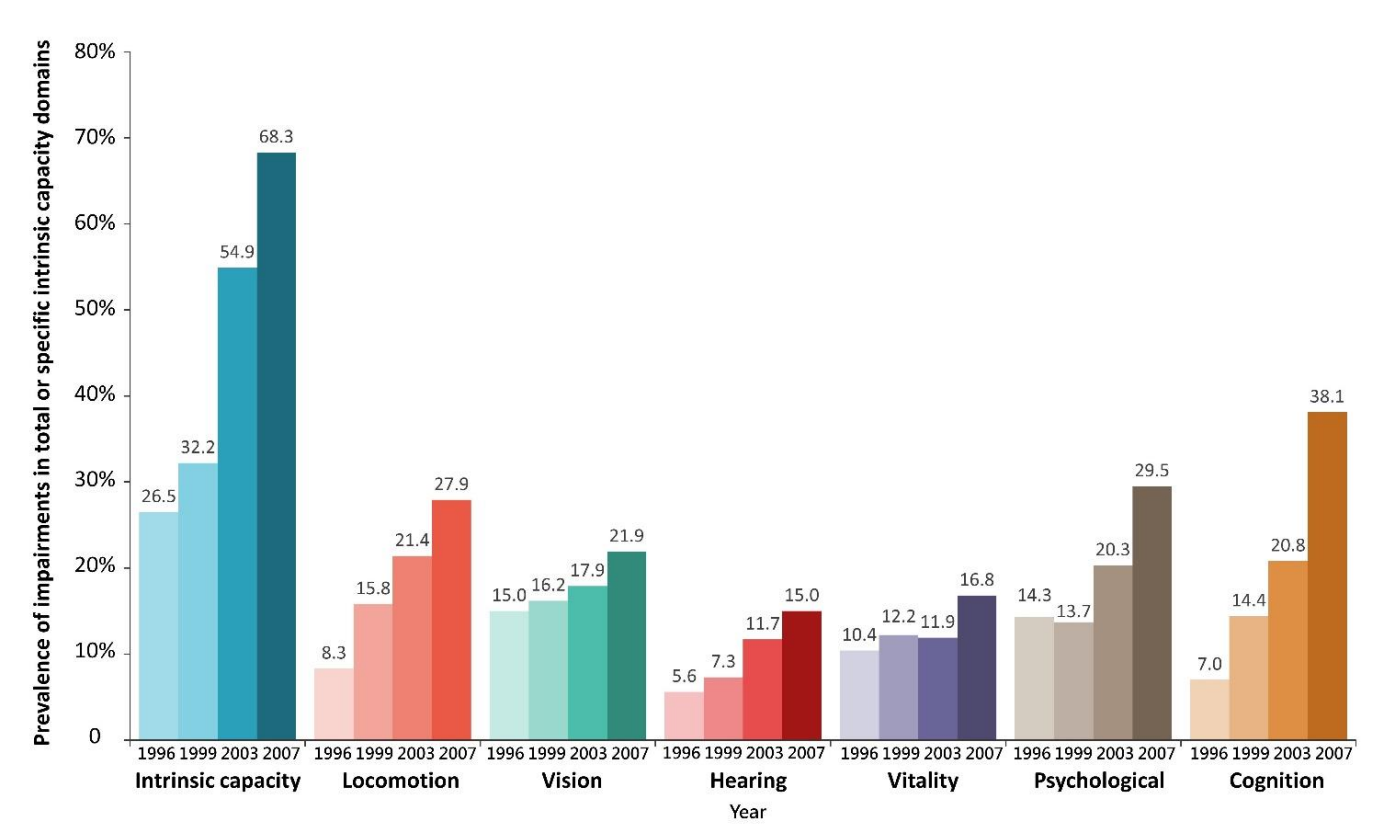
Prevalence of intrinsic capacity impairments by different domains during the trajectory period.

Out of the 6,736 participants included in the third to sixth waves of the TLSA survey, a total of 1,782 eligible participants were selected for the present study. Figure 2 depicts the prevalence trends of IC impairments across different domains during a 12-year trajectory period. It is evident that the overall prevalence of impairments in the six components of IC exhibited a substantial increase over time. Notably, cognitive impairments experienced the most pronounced rise, with a prevalence rate of 7.01% in 1996 escalating to 38.10% in 2007. Similarly, locomotion impairments demonstrated a significant increase, from 8.27% to 27.95% during the same period.

### Subtypes of IC decline

**Figure 3.**
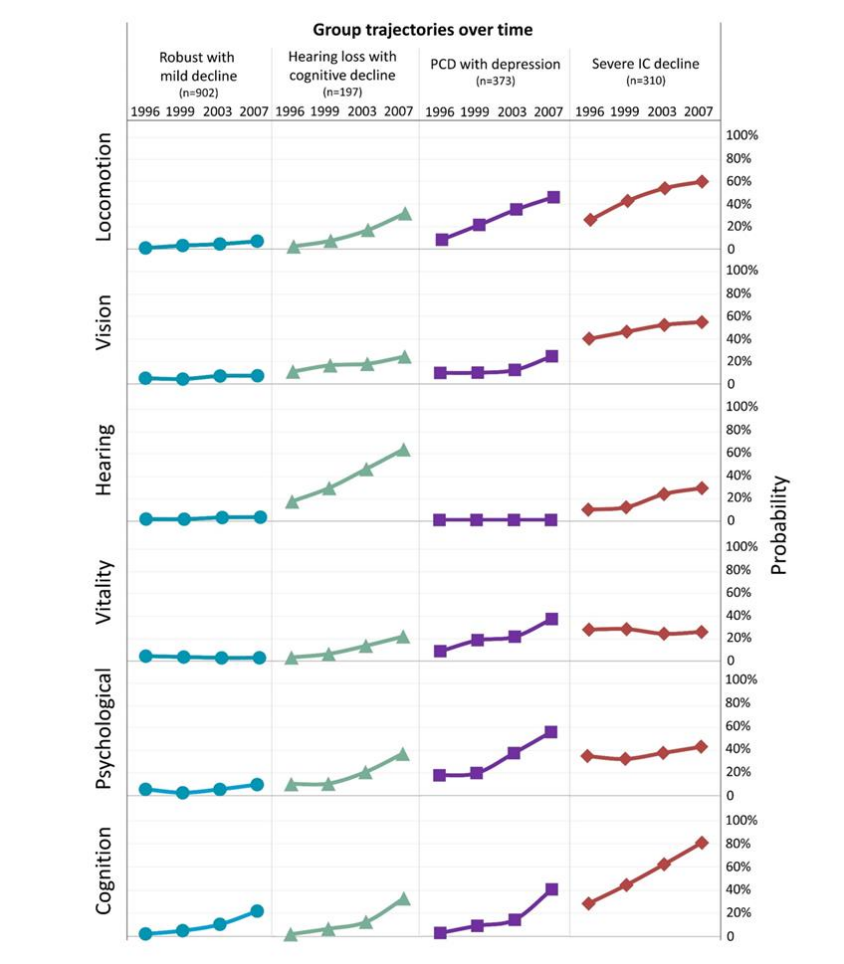
Results of group-based multitrajectory modelling: intrinsic capacity decline subtypes.

After applying the model selection procedure, it was determined that the four-trajectory model outperformed models with fewer trajectories (BIC values: four-trajectory model = -14844.91; three-trajectory model = -14902.47; two-trajectory model = -15036.11), while the five-trajectory model exhibited instability. Consequently, the four-trajectory model was selected as the final one for subsequent analyses. Following the model-building process, participants were assigned to the trajectory with the highest membership probability, as determined by their posterior probabilities of membership in multiple trajectories (Fig. 3).

The four decline subtypes were labelled as follows: "robust with mild decline," "hearing loss with cognitive decline," "physio-cognitive decline (PCD) with depression," and "severe IC decline." The "robust with mild decline" subtype accounted for 50.6% (n=902) of the participants, indicating a relatively healthy population. Approximately 11.1% (n=197) of participants were classified as "hearing loss with cognitive decline," while 20.9% (n=373) exhibited concurrent physical and cognitive declines with depression, falling into the "PCD with depression" category. The "severe IC decline" subtype, characterized by high probabilities of impairments across all IC domains - mostly notably a consistently high rate of visual impairment - was represented by 17.4% (n=310) of participants.

Significant variations in baseline characteristics were observed among participants with different subtypes of IC decline (Table 1). The "hearing loss with cognitive decline" group had a higher proportion of males (p < 0.01) and exhibited a greater prevalence of comorbid conditions, including hypertension, diabetes mellitus, and hyperlipidaemia (p < 0.01), compared to other groups. The "PCD with depression" group reported the lowest QoL scores (p < 0.01) and the highest levels of depressive symptoms (p < 0.01). Participants in the "severe IC decline" group were significantly older (p < 0.01), scored lower on the SPMSQ (p < 0.01), and demonstrated a higher likelihood of disabilities (p < 0.01) compared to other subtypes.

**Table 1:** Baseline characteristics of intrinsic capacity decline subtypes.

Mean ± SD, or number (%)	Total	Robust with mild decline	Hearing loss with cognitive decline	PCD with depression	Severe IC decline	P value
**Number**	1,782	902	197	373	310	
**Sex (Female)**	865 (48.5)	337 (37.4)	62 (31.5)	219 (58.7)	247 (79.7)	<0.01
**Age (Year)**						
**65-69**	106 (6.0)	67 (7.4)	7 (3.6)	24 (6.4)	8 (2.6)	<0.01
**70-74**	460 (25.8)	278 (30.8)	35 (17.8)	114 (30.6)	33 (10.7)	
**75-79**	505 (28.3)	266 (29.5)	54 (27.4)	107 (28.7)	78 (25.2)	
**80-84**	475 (26.7)	213 (23.6)	60 (30.5)	85 (22.8)	117 (37.7)	
**85+**	236 (13.2)	78 (8.7)	41 (20.8)	43 (11.5)	74 (23.9)	
**The level of education**						
**No schooling**	654 (36.7)	216 (24.0)	67 (34.0)	133 (35.7)	238 (76.8)	<0.01
**Elementary**	695 (39.0)	384 (42.6)	84 (42.6)	168 (45.0)	59 (19.0)	
**Junior/Senior High**	319 (17.9)	225 (24.9)	30 (15.2)	53 (14.2)	11 (3.6)	
**College/Graduate school**	114 (6.4)	77 (8.5)	16 (8.1)	19 (5.1)	2 (0.7)	
**Smoking**	261 (14.7)	162 (18.0)	30 (15.2)	47 (12.6)	22 (7.1)	<0.01
**Alcohol use**	395 (22.2)	260 (28.8)	42 (21.3)	72 (19.3)	21 (6.8)	<0.01
**Visual acuity**						
**Very clear**	222 (12.5)	162 (18.0)	15 (7.6)	35 (9.4)	10 (3.2)	<0.01
**Clear**	797 (44.7)	485 (53.8)	89 (45.2)	156 (41.8)	67 (21.6)	
**Average**	372 (20.9)	186 (20.6)	46 (23.4)	80 (21.5)	60 (19.4)	
**Poor**	351 (19.7)	67 (7.4)	45 (22.8)	94 (25.2)	145(46.8)	
**Very poor**	40 (2.2)	2 (0.2)	2 (1.0)	8 (2.1)	28 (9.0)	
**Hearing ability**						
**Very clear**	410 (23.0)	288 (31.9)	9 (4.6)	84 (22.5)	29 (9.3)	<0.01
**Clear**	790 (44.3)	441 (48.9)	18 (9.1)	206 (55.2)	125 (40.3)	
**Average**	314 (17.6)	144 (16.0)	22 (11.2)	83 (22.3)	65 (21.0)	
**Poor**	260 (14.6)	29 (3.2)	144 (73.1)	0 (0)	87 (28.1)	
**Very poor**	8 (0.5)	0 (0)	4 (2.0)	0 (0)	4 (1.3)	
**ADL (0-6)**	5.5 ± 1.3	6.0 ± 0.4	5.4 ± 1.5	5.2 ± 1.7	4.8 ± 2.0	<0.01
**IADL (0-6)**	4.5 ± 1.8	5.4 ± 1.1	4.2 ± 1.9	4.0 ± 1.8	2.7 ± 1.9	<0.01
**Mobility performance (0-9)**	5.7 ± 2.7	7.2 ±2.0	5.2 ± 2.6	4.2 ± 2.5	3.4 ± 2.3	<0.01
**SPMSQ (0-9)**	7.4 ± 1.9	8.1 ± 1.4	7.7 ± 1.5	7.4 ± 1.7	5.2 ± 2.1	<0.01
**CES-D (0-30)**	5.6 ± 6.1	2.6 ± 3.3	6.8 ± 6.0	10.8 ± 6.9	8.3 ± 6.9	<0.01
**Quality of life (0-12)**	8.1 ± 2.9	9.2 ± 2.3	8.1± 2.9	6.7 ± 3.2	7.0 ± 3.1	<0.01
**Comorbidities**						
**Hypertension**	867 (48.7)	401 (44.5)	112 (56.9)	191 (51.2)	163 (52.6)	<0.01
**Diabetes mellitus**	314 (17.6)	126 (14.0)	48 (24.4)	83 (22.3)	57 (18.4)	<0.01
**Hyperlipidemia**	276 (15.5)	134 (14.9)	38 (19.3)	64 (17.2)	40 (12.9)	0.19
**Heart disease**	480 (26.9)	196 (21.7)	62 (31.5)	133 (35.7)	89 (28.7)	<0.01
**Stroke**	115 (6.5)	36 (4.0)	21 (10.7)	28 (7.5)	30 (9.7)	<0.01
**Cancer**	76 (4.3)	35 (3.9)	12 (6.1)	23 (6.2)	6 (1.9)	0.02
**COPD or asthma**	232 (13.0)	81 (9.0)	39 (19.8)	73 (19.6)	39 (12.6)	<0.01
**Arthritis**	417 (23.4)	143 (15.9)	42 (21.3)	128 (34.3)	104 (33.6)	<0.01
**Gastric ulcer**	394 (22.1)	175 (19.4)	48 (24.4)	105 (28.2)	66 (21.3)	<0.01
**Hepatobiliary disease**	169 (9.5)	75 (8.3)	29 (14.7)	93 (10.5)	26 (8.4)	0.04
**Cataract**	893 (50.1)	417 (46.2)	108 (54.9)	185 (49.6)	183 (59.0)	<0.01
**Chronic kidney disease**	189 (10.6)	71 (7.9)	29 (14.7)	51 (13.7)	38 (12.3)	<0.01
**Gout**	186 (10.4)	88 (9.8)	19 (9.6)	48 (12.9)	31 (10.0)	0.39
**Osteoporosis**	276 (15.5)	134 (14.9)	38 (19.3)	64 (17.2)	40 (12.9)	0.18
**Multimorbidity**						
**0**	214 (12.0)	149 (16.5)	17 (8.6)	24 (6.4)	24 (7.7)	<0.01
**1**	319 (17.9)	197 (21.8)	23 (11.7)	49 (13.1)	50 (16.1)	
**2**	418 (23.5)	216 (24.0)	43 (21.8)	85 (22.8)	74 (23.9)	
≥**3**	831 (46.6)	340 (37.7)	114 (57.9)	215 (57.6)	162 (52.3)	

SD, standard deviation; PCD, physio-cognitive decline; SPMSQ, Short Portable Mental State Questionnaire; CES-D, Center for Epidemiologic Studies Depression; ADL, activities of daily living; IADL, instrumental activities of daily living; COPE, chronic obstructive pulmonary disease

### Risk of healthy ageing-related outcomes

#### Decline in ADLs

The association between subtypes of IC decline and the risk of decline in ADLs over the 4-year and 8-year durations is summarized in Table 2. During the 4-year period, participants in the "PCD with depression" group (aOR 2.61; 95% CI, 1.81-3.75; p < 0.01) and the "severe IC decline" group (aOR 2.26; 95% CI, 1.46-3.47; p < 0.01) exhibited a higher likelihood of decline in ADLs compared to the "robust with mild decline" group. Over the long-term, participants in the "hearing loss with cognitive decline" group (aOR 1.80; 95% CI, 1.08-3.02; p = 0.03), the "PCD with depression" group (aOR 2.08; 95% CI, 1.36-3.20; p < 0.01), and the "severe IC decline" group (aOR 4.55; 95% CI, 2.63-7.88; p < 0.01) all demonstrated a higher risk of decline in ADLs.

#### Decline in IADLs

All three subtypes of IC decline were found to be significantly associated with an increased risk of decline in IADLs (all p<0.01). After a 4-year follow-up, participants in the "severe IC decline" group had the highest risk of decline in IADLs (aOR 2.26; 95% CI, 1.47-3.47; p < 0.01). Similarly, at an 8-year follow-up, both the "PCD with depression" and "severe IC decline" groups were associated with the highest risk of decline in IADLs (Table 2).

**Table 2:** Association of subtypes in intrinsic capacity decline and age-related outcomes.

	4-year			8-year		
	aOR/aHR	95% CI	P-value	aOR/aHR	95% CI	P-value
**Decline in ADL**						
**Robust with mild decline**	*ref*	-	-	*ref*	-	-
**Hearing loss with cognitive decline**	1.21	0.75-1.95	0.45	1.80	1.08-3.02	0.03
**PCD with depression**	2.61	1.81-3.75	<0.01	2.08	1.36-3.20	<0.01
**Severe IC decline**	2.26	1.46-3.47	<0.01	4.55	2.63-7.88	<0.01
**Decline in IADL**						
**Robust with mild decline**	*ref*	-	-	*ref*	-	-
**Hearing loss with cognitive decline**	1.95	1.30-2.93	<0.01	2.23	1.31-3.79	<0.01
**PCD with depression**	1.86	1.34-2.58	<0.01	3.44	2.14-5.54	<0.01
**Severe IC decline**	2.26	1.47-3.47	<0.01	3.52	1.85-6.72	<0.01
**Reduced mobility performance**						
**Robust with mild decline**	*ref*	-	-	*ref*	-	-
**Hearing loss with cognitive decline**	1.70	1.12-2.58	0.01	1.70	1.02-2.85	0.04
**PCD with depression**	1.74	1.22-2.48	<0.01	2.04	1.31-3.18	<0.01
**Severe IC decline**	1.87	1.22-2.87	<0.01	2.30	1.30-4.07	<0.01
**Cognitive decline**						
**Robust with mild decline**	*ref*	-	-	*ref*	-	-
**Hearing loss with cognitive decline**	1.21	0.80-1.83	0.36	1.39	0.84-2.29	0.20
**PCD with depression**	1.19	0.86-1.65	0.39	1.45	0.96-2.18	0.08
**Severe IC decline**	2.04	1.31-3.17	<0.01	1.94	1.07-3.50	0.03
**Fall**						
**Robust with mild decline**	*ref*	-	-	*ref*	-	-
**Hearing loss with cognitive decline**	1.00	0.65-1.53	0.99	1.09	0.66-1.79	0.75
**PCD with depression**	1.63	1.18-2.25	<0.01	0.90	0.60-1.36	0.62
**Severe IC decline**	1.42	0.97-2.08	0.07	1.12	0.66-1.90	0.67
**Reduced quality of life**						
**Robust with mild decline**	*ref*	-	-	*ref*	-	-
**Hearing loss with cognitive decline**	2.53	1.66-3.87	<0.01	3.46	2.01-5.96	<0.01
**PCD with depression**	1.63	1.16-2.29	<0.01	1.94	1.25-3.02	<0.01
**Severe IC decline**	2.14	1.42-3.23	<0.01	5.45	3.00-9.91	<0.01
**Mortality**						
**Robust with mild decline**	*ref*	-	-	*ref*	-	-
**Hearing loss with cognitive decline**	1.30	0.90-1.88	0.16	1.01	0.80-1.28	0.93
**PCD with depression**	1.67	1.22-2.29	<0.01	1.58	1.31-1.91	<0.01
**Severe IC decline**	2.36	1.70-3.28	<0.01	1.98	1.61-2.44	<0.01

ADL, activities of daily living; IADL, instrumental activities of daily living; PCD, physio-cognitive decline; OR, odds ratio; HR, hazards ratio; CI, confidence interval OR for decline in ADL, decline in IADL, reduced mobility performance, cognitive decline, fall, reduced quality of life; HR for mortality. Adjust for age, sex, education, smoking, alcohol use, hypertension, diabetes mellitus, heart disease, stroke, cancer, COPD or asthma, arthritis, gastric ulcer, hepatobiliary disease, cataract, chronic kidney disease, multimorbidity, and baseline assessment (except for mortality and fall).

#### Reduced mobility performance

After a 4-year follow-up, participants in the "hearing loss with cognitive decline" (aOR 1.70; 95% CI, 1.12-2.58; p = 0.01), "PCD with depression" (aOR 1.74; 95% CI, 1.22-2.48; p < 0.01), and "severe IC decline" groups (aOR 1.87; 95% CI, 1.22-2.87; p < 0.01) had comparable increased risks of reduced mobility performance compared to the "robust with mild decline" group. However, at an 8-year follow-up, the "severe IC decline" group exhibited the highest risk of reduced mobility performance (aOR 2.30; 95% CI, 1.30-4.07; p < 0.01) (Table 2).

#### Cognitive decline

In relation to cognitive decline, unlike other outcome indicators, it was found that only the "severe IC decline" group exhibited an increased risk at both the 4-year follow-up (aOR 2.04; 95% CI, 1.31-3.17; p < 0.01) and the 8-year follow-up (aOR 1.94; 95% CI, 1.07-3.50; p = 0.03) (Table 2).

#### Falls

Participants belonging to the "PCD with depression" group exhibited a higher risk of falls during the 4-year period in comparison to the "robust with mild decline" group (aOR 1.63; 95% CI, 1.18-2.25; p < 0.01). However, this association did not reach statistical significance during the 8-year period (aOR 0.90; 95% CI, 0.60-1.36; p=0.62) (Table 2).

#### Quality of life

Participants in subtypes other than the "robust with mild decline" group exhibited a significantly higher risk of experiencing reduced QoL over both the 4-year and 8-year periods. Specifically, the "hearing loss with cognitive decline" group showed the greatest risk of reduced QoL during the 4-year period, with a notable 2.5-fold increase (aOR 2.53; 95% CI, 1.66-3.87; p<0.01). Similarly, over the 8-year period, participants in the "severe IC decline" group faced the highest risk of reduced QoL, demonstrating a substantial 5.47-fold increase (aOR 5.45; 95% CI, 3.00-9.91; p<0.01) (Table 2).

#### Mortality

The association between subtypes of IC decline and age-related outcomes is presented in Table 2. The "PCD with depression" group (aHR 1.67; 95% CI, 1.22-2.29; p < 0.01) and the "severe IC decline" group (aHR 2.36; 95% CI, 1.70-3.28; p < 0.01) exhibited a significantly increased risk of 4-year mortality compared to the "robust with mild decline" group. Similar patterns were observed for 8-year mortality, with a 1.6-fold increase (aHR=1.58; 95% CI, 1.31-1.91; p < 0.01) in the "PCD with depression" group and a 2.0-fold increase (aHR 1.98; 95% CI, 1.61-2.44; p < 0.01) in the "severe IC decline" group.

## DISCUSSION

This novel longitudinal cohort study employed a data-driven approach to investigate distinct subtypes of IC decline and their associations with adverse outcomes in the older population. The study identified four subtypes: robust with mild decline, hearing loss with cognitive decline, PCD with depression, and severe IC decline. Each subtype had varying effects on age-related outcomes during long-term follow-up. The "severe IC decline" subtype exhibited the highest risk for declines in ADLs, IADLs, cognition, reduced QoL, and mortality, excluding falls. Individuals in this category may experience mobility limitations that paradoxically reduce the risk of falls. The risks associated with the "severe IC decline" subtype were amplified at the 8th year compared to the 4th year, suggesting a cumulative effect. Despite variations in the measurement of each IC domain, our study consistently revealed patterns of IC decline across domains, as supported by a recent latent class analysis [7]. Notwithstanding differences in participant characteristics, study settings, cohorts, and analytical methods, the identified subtypes of IC decline demonstrate notable similarities across these studies. This convergence strengthens the notion that these specific subtypes hold mechanistic importance, providing insights into the underlying processes associated with IC decline. Both studies intriguingly highlight the significance of the cognition domain within the IC framework, as it consistently emerges across diverse subtypes.

Given the potential involvement of shared neuro-pathologies like Alzheimer's disease, Parkinson's disease, and cerebrovascular disease, as well as mechanisms related to the muscle-brain axis and chronic inflammation [8], it is plausible that neurodegeneration plays a role in these associations. In this study, around 20% of participants were classified as "PCD with depression", indicating a prevalent combination of physical and mental impairments. The association between PCD and depression has been previously reported in various studies [25, 26]. Depression, potentially an early sign of neurodegenerative disorder, could arise from chronic inflammation that impacts central dopaminergic function [27]. Furthermore, a recent longitudinal study found a correlation between elevated levels of inflammatory markers (GDF-15 and MCP-1) and impairment in the psychological aspects of IC [28]. A recent study identified a novel neurocircuit linking the cerebellum, amygdala, and hippocampus in older individuals diagnosed with physio-cognitive decline syndrome (PCDS) [29]. Notably, this neurocircuit was also confirmed in a mouse model, further validating the findings [30]. The identification of the "PCD with depression" subtype in this study provides additional support for previous research findings [8, 30]. Evidence from well-designed randomized controlled trials has demonstrated that despite the association of PCDS with an increased risk of disability, dementia, and mortality, multidomain interventions have shown potential reversibility, thereby emphasising the preventive and reversible nature of PCDS [31]. Positive results were found in a study examining the impact of incorporating a multidomain intervention into healthcare on QoL [32], highlighting the significance of a personalized approach within the public health strategy for promoting healthy ageing in the community.

This study identified an additional subtype, "hearing loss with cognitive decline," which was more common in males and had a higher prevalence of comorbid conditions, particularly among individuals aged 80 and older, suggesting its impact on the oldest-old population; this finding aligns with previous research indicating potential shared underlying age-related neuro-degenerative mechanisms between hearing loss and cognitive decline [33]. Moreover, the 'cascade hypothesis' suggests that hearing loss in older adults can directly affect cognition through reduced auditory input or indirectly through social isolation and depression [34]. The "hearing loss with cognitive decline" subtype was associated with a 2.5-fold higher risk of reduced QoL over a 4-year period, even after adjusting for baseline QoL. Age-related hearing loss is a common sensory impairment among older individuals and can significantly impact their social, functional, and psychological well-being. The combination of hearing loss and cognitive impairment may have a synergistic negative effect on QoL. However, the use of assistive hearing devices like hearing aids and cochlear implants may improve hearing ability and potentially enhance cognitive function, thereby maintaining QoL in this subtype, although intervention studies are needed to confirm this [35]. The identification of the "severe IC decline" subtype in our study aligns with the WHO's policy framework for healthy ageing, which categorises individuals into subpopulations based on their level of IC: those with high and stable IC, those with declining IC, and those with significant IC losses.^2^ This finding is consistent with previous studies that have reported similar longitudinal patterns of IC [36, 37].

This study possesses multiple strengths, including its 20-year longitudinal design with a nationally representative sample and high survey response rate, ensuring external validity. Additionally, the utilization of a group-based multi-trajectory model analysis strengthens the evidence on IC decline subtypes across domains. Lastly, the study comprehensively evaluated age-related outcomes and offers valuable insights for prioritizing clinical interventions in promoting healthy ageing. Our findings also provide comprehensive insights into the patterns of decline in IC across various domains, offering robust evidence to support the prioritization of clinical interventions and the implementation of functional maintenance strategies. Despite extensive research efforts, this study has some limitations. First, the self-reported difficulties in IC components may not perfectly reflect the actual status and the observational nature of the study may suggest that the identified associations between subtypes of IC decline and aging-related outcomes do not necessarily imply causality. In addition, changes in healthcare systems, societal factors, and technological advancements during our longitudinal study period may have potential impacts on our findings. However, the consistent trends observed in this study compared to other countries support the robustness of our findings suggest that the findings remain valuable^40^. Second, the inclusion of data from participants aged 65 years old and older who completed at least two waves of the TLSA surveys from 1996 to 2007 may introduce a potential health participant bias, limiting the generalizability of the findings. Third, while the naming of subtypes of IC decline is based on the outputs of GBMTM, some subtypes of IC decline, particularly PCD, are externally validated in numerous studies conducted in different countries [7, 8, 31], such as Japan. Future research using the same GBMTM approach, but different data sources is warranted to further validate our findings. Fourth, given the limitations of questionnaire data, this study lacks comprehensive information pertaining to aging biomarkers, clinical condition management, and healthcare resource utilization. Future research endeavors should adopt a more comprehensive and interdisciplinary approach, encompassing biological and functional aging, disease progression and treatment, as well as psychosocial ramifications. This approach is vital for the development of effective strategies aimed at promoting healthy aging and extending healthy longevity. Last but not the least, although the use of a decline of at least one point to define a reduction in QoL may not be considered clinically meaningful, this study revealed a significantly increased risk of reduced QoL over 4 and 8-year periods across all subtypes of IC decline compared to the robust with mild decline group, while controlling for baseline QoL, and provided valuable longitudinal insights into the association between subtypes of IC decline and QoL fluctuations.

In summary, our study has distinctly delineated subtypes of IC impairment and elucidated their associated adverse outcomes, which hold several clinical and policy implications. First, the incorporation of IC and impairment subtypes into the management of chronic conditions is imperative. Elements of IC traditionally regarded as mere consequences of aging, affecting mobility, cognition, vitality, sensory functions, and psychosocial well-being, should receive heightened clinical attention within primary healthcare systems, as well as acute care settings. Second, primary healthcare systems should implement effective intervention programs designed to ameliorate IC impairments. Multidomain interventions encompassing exercise, cognitive stimulation, nutritional interventions, social participation, and the management of chronic conditions should be introduced [31, 32, 38]. Third, subsequent research in the context of population aging should take into account IC, its subtypes, considering their type-specific outcomes and impacts.

## Conclusion

This study elucidated four distinct subtypes of IC decline, namely robust with mild decline, hearing loss with cognitive decline, physio-cognitive decline with depression, and severe IC decline. The findings substantiate the clustering of IC domains over time and demonstrate the discrete impacts of each subtype on various age-related outcomes, encompassing functional limitations, restricted mobility, cognitive impairment, falls, diminished QoL, and mortality. Hence, incorporating person-centric principles becomes imperative in the public health strategy to foster healthy ageing within the community.
